# *Bacteroides fragilis* Acute Hematogenous Osteomyelitis in a Young Female with Sickle Cell Disease

**DOI:** 10.1155/2023/6340222

**Published:** 2023-12-05

**Authors:** Nada Bassam Hamieh, Hiba Abdul Hamid Abou Layla, Rola Ali Ali, Zeina Bayram, Abdul Rahman Bizri

**Affiliations:** ^1^American University of Beirut Medical Center, Beirut, Lebanon; ^2^American University of Beirut, Beirut, Lebanon

## Abstract

Patients with sickle cell disease are at increased risk for multiple infections including osteomyelitis. The most reported causative organisms are *Salmonella* spp. and *Staphylococcus aureus.* Anaerobic infections including *Bacteroides fragilis* are not commonly seen. Here, we report the first case of a 28-year-old female patient with sickle cell disease and acute hematogenous *Bacteroides fragilis* tibial osteomyelitis. Diagnosis was made by isolating the organism from blood and tibial fluid cultures. The patient was successfully managed with a course of intravenous followed by oral antibiotics and percutaneous drainage of collection and responded well. This case report will shed light on the importance of *Bacteroides fragilis* as a causative organism for osteomyelitis in sickle cell disease patients, thereby affecting the management of these patients.

## 1. Background

Sickle cell disease (SCD) patients are more prone to developing osteoarticular infections due to multiple risk factors including functional hyposplenism, impaired phagocytosis, and defective complement activity [1, 2]. Another major risk factor is bone ischemia and necrosis due to several vaso-occlusive episodes [3]. *Salmonella* spp. followed by *Staphylococcus aureus* are the most described bacterial etiologies of osteomyelitis in patients with SCD, whereas anaerobic bacteria are uncommonly encountered [4–6]. Herein, we report a case of left lower limb (tibial) osteomyelitis in a 28-year-old female with sickle cell disease caused by *Bacteroides fragilis.*

## 2. Case Presentation

A 28-year-old female patient known to have sickle cell disease since childhood maintained on hydroxyurea and folic acid with a history of multiple pain crises presented to our emergency department for a fever reaching 38.5°C of two days duration and left lower extremity pain of five days prior to presentation. On the physical exam, she was alert and oriented. She had a temperature of 39°C and stable blood pressure. The respiratory exam revealed normal breath sounds. The abdomen was soft, nontender with a splenomegaly of 4 cm below the costal margin. The left lower leg exam was pertinent for a mildly ill-defined erythematous area of 5 cm in diameter and edema below the left knee.

Initial laboratory evaluation revealed a total white count of 4300/mm^3^ with neutrophils of 91%, hemoglobin of 7.5 mg/dL, and C-reactive protein (CRP) of 154 mg/L. Urine analysis was positive for white blood cells and bacteria. A chest X-ray was done and revealed no consolidations or effusions. Blood and urine cultures were taken, and the patient was started initially on cefotaxime for suspected urinary tract infection. She was also given intravenous (IV) fluids and analgesics, as her left leg pain was attributed to a sickle cell crisis. Gram-negative rods grew in one out of two blood culture bottles after 22 hours. A computed tomography scan of the abdomen and pelvis with intravenous contrast was performed. It showed moderate-to-severe splenomegaly of 20 cm with multiple subcapsular hypodensities suggestive of small infarcts; no intraabdominal collection was detected. *Bacteroides fragilis* was identified as the causative agent of her bacteremia, and therapy subsequently escalated to meropenem. The patient's left leg's pain meanwhile increased, as well as the erythema and tenderness of anterior shin. Magnetic resonance imaging of the same extremity with and without gadolinium was done and revealed extensive enhancing edema of the superficial and deep planes of the leg with rim enhancing fluid collection in the anterior proximal compartment, abutting the adjacent tibial lateral cortex with associated significant periosteal enhancement, representing a soft tissue abscess with adjacent tibial osteomyelitis. 13 mL of pus were aspirated under radiologic guidance and specimen was sent to cytology and culture. Fluid culture also revealed *Bacteroides fragilis* establishing the diagnosis of *Bacteroides fragilis* bacteremia with acute hematogenous osteomyelitis. The patient continued a 10-day course of meropenem in the hospital as the susceptibility spectrum of anaerobic bacteria was not available in our institution. She was then charged. Her treatment included intravenous metronidazole followed by oral metronidazole administration for a total duration of 6 weeks. Follow up visits in the clinic revealed resolution of left leg pain and tenderness with decrease in CRP to 8 mg/L.

Sickle cell disease is a risk factor for osteomyelitis and septic arthritis where osteomyelitis is present at a rate of 18% and septic arthritis at a rate of 7% in sickle cell disease [7]. The most common causative agents are *Salmonella* spp. and *Staphylococcus aureus* with anaerobes, generally *Bacteroides fragilis*, isolated in less than 1% of acute hematogenous osteomyelitis [8]. *Bacteroides fragilis* is a nonspore forming, nonmotile anaerobic Gram-negative bacilli that resides in the gastrointestinal tract. It is associated with many human diseases (diarrhea, bacteremia, pelvic infections, meningitis…) and can infrequently cause osteomyelitis in SCD patients [9, 10].

In SCD, the gut mucosa is compromised due to intravascular sickling, which leads to translocation of the gut flora into the blood stream. As the reticuloendothelial system and immune response in SCD patients are compromised, persistent bacteremia is frequently encountered. The bacteria will reside in areas of sluggish blood circulation such as the metaphysis of long bone, leading to osteomyelitis. Consequently, the metaphysis of long bones in SCD is a site of infarct, which further provides a suitable environment for the anaerobes [11].

In our search through the current literature using PubMed, Embase, and Google Scholar data bases, *Bacteroides fragilis* osteomyelitis in SCD was reported in only six patients; the bones affected were the humerus in one case, three cases involving the vertebrae along with bilateral psoas abscesses in one of them, and only one case of tibial osteomyelitis associated with knee septic arthritis [8, 11–14]. All cases were children, except for a 23-year-old woman who presented with femoral osteomyelitis [12].

Our patient was diagnosed with left tibial osteomyelitis due to *Bacteroides fragilis* associated with secondary bacteremia. To our knowledge, this is the first reported case of *Bacteroides fragilis* tibial osteomyelitis in a young adult with SCD.

Treatment of *Bacteroides fragilis* osteomyelitis in SCD patients includes symptomatic management, hydration, antibiotic therapy tailored according to the susceptibility profile, adequate drainage of abscesses, and soft tissue collections if needed [15]. Currently, *Bacteroides fragilis* is demonstrating increased resistance to most antibiotics; the suggested mechanism of resistance could be related to bacterial multidrug efflux pumps [16]. *Bacteroides* have demonstrated resistance to penicillin and ampicillin with increasing resistance to clindamycin; however, metronidazole, carbapenems, and beta-lactam/beta-lactam inhibitors retain good activity against *B. fragilis*, and resistance remains uncommon against these compounds [17, 18]. Susceptibility testing against anaerobes was not available at our institution. She received meropenem followed by metronidazole that was continued upon discharge and had an excellent clinical and laboratory response. Surgical drainage of abscesses or soft tissue collections might be required for diagnostic and therapeutic purposes to achieve full recovery [8]. Our patient required drainage of soft tissue collection for both diagnostic and therapeutic purposes.

## 3. Conclusion

In conclusion, we are reporting the first case of *Bacteroides fragilis* acute hematogenous tibial osteomyelitis in a young adult with SCD. *Bacteroides fragilis* is an uncommon pathogen, and osteomyelitis caused by these bacteria is rarely reported in SCD patients.

## Data Availability

All data present in this study are available from the corresponding author upon a reasonable request.
